# The association of cognitive functioning as measured by the DemTect with functional and clinical characteristics of COPD: results from the COSYCONET cohort

**DOI:** 10.1186/s12931-019-1217-5

**Published:** 2019-11-14

**Authors:** Sarah Marietta von Siemens, Robert Perneczky, Claus F. Vogelmeier, Jürgen Behr, Diego Kauffmann-Guerrero, Peter Alter, Franziska C. Trudzinski, Robert Bals, Christian Grohé, Sandra Söhler, Benjamin Waschki, Johanna I. Lutter, Tobias Welte, Rudolf A. Jörres, Kathrin Kahnert, the COSYCONET study group, Stefan Andreas, Robert Bals, Jürgen Behr, Kathrin Kahnert, Burkhard Bewig, Roland Buhl, Ralf Ewert, Beate Stubbe, Manfred Gogol, Christian Grohé, Rainer Hauck, Matthias Held, Berthold Jany, Markus Henke, Felix Herth, Gerd Höffken, Hugo A. Katus, Anne-Marie Kirsten, Henrik Watz, Rembert Koczulla, Klaus Kenn, Juliane Kronsbein, Cornelia Kropf-Sanchen, Christoph Lange, Peter Zabel, Michael Pfeifer, Winfried J. Randerath, Werner Seeger, Michael Studnicka, Christian Taube, Helmut Teschler, Hartmut Timmermann, J. Christian Virchow, Claus Vogelmeier, Ulrich Wagner, Tobias Welte, Hubert Wirtz

**Affiliations:** 1 Institute and Outpatient Clinic for Occupational, Social and Environmental Medicine, Comprehensive Pneumology Center Munich (CPC-M), Ludwig-Maximilians-Universität München, Ziemssenstr 1, 80336 Munich, Germany; 2 Department of Psychiatry and Psychotherapy, University Hospital, LMU Munich, Munich, Germany; 3 German Center for Neurodegenerative Disorders (DZNE) Munich, Munich, Germany; 4 Munich Cluster for Systems Neurology (SyNergy), Munich, Germany; 5 Ageing Epidemiology Research Unit (AGE), School of Public Health, Imperial College London, London, UK; 6 Department of Medicine, Pulmonary and Critical Care Medicine, University Medical Center Giessen and Marburg, Philipps-University Marburg, Member of the German Center for Lung Research (DZL), Baldingerstrasse, 35043 Marburg, Germany; 7 Department of Internal Medicine V, University of Munich (LMU), Comprehensive Pneumology Center, Member of the German Center for Lung Research, Ziemssenstr. 1, 80336 Munich, Germany; 8 Asklepios Fachkliniken München-Gauting, Robert-Koch-Allee 2, 82131 Gauting, Germany; 9 Department of Internal Medicine V – Pulmonology, Allergology, Respiratory Intensive Care Medicine, Saarland University Hospital, Kirrberger Straße 1, 66424 Homburg, Germany; 10 Evangelische Lungenklinik, Lindenberger Weg 27, 13125 Berlin, Germany; 11 ASCONET Study Coordination Office, University of Marburg, Baldingerstraße, 35043 Marburg, Germany; 12 Department of General and Interventional Cardiology, University Heart Center Hamburg, Hamburg, Germany; 13 LungenClinic Grosshansdorf, Airway Research Center North (ARCN), Member of the German Center for Lung Research (DZL), Grosshansdorf, Germany; 14 Institute of Health Economics and Health Care Management, Helmholtz Zentrum München GmbH – German Research Center for Environmental Health, Comprehensive Pneumology Center Munich (CPC-M), Member of the German Center for Lung Research, Ingolstädter Landstr. 1, 85764 Munich, Germany; 15 Department of Pneumology, Hannover Medical School, Carl-Neuberg-Str. 1, 30625 Hannover, Germany

**Keywords:** COPD, Dementia, Cognitive impairment

## Abstract

Alterations of cognitive functions have been described in COPD. Our study aimed to disentangle the relationship between the degree of cognitive function and COPD characteristics including quality of life (QoL).

Data from 1969 COPD patients of the COSYCONET cohort (GOLD grades 1–4; 1216 male/ 753 female; mean (SD) age 64.9 ± 8.4 years) were analysed using regression and path analysis. The DemTect screening tool was used to measure cognitive function, and the St. George‘s respiratory questionnaire (SGRQ) to assess disease-specific QoL.

DemTect scores were < 9 points in 1.6% of patients and < 13 points in 12% when using the original evaluation algorithm distinguishing between < 60 or > =60 years of age. For statistical reasons, we used the average of both algorithms independent of age in all subsequent analyses. The DemTect scores were associated with oxygen content, 6-min-walking distance (6-MWD), C-reactive protein (CRP), modified Medical Research Council dyspnoea scale (mMRC) and the SGRQ impact score. Conversely, the SGRQ impact score was independently associated with 6-MWD, FVC, mMRC and DemTect. These results were combined into a path analysis model to account for direct and indirect effects. The DemTect score had a small, but independent impact on QoL, irrespective of the inclusion of COPD-specific influencing factors or a diagnosis of cognitive impairment.

We conclude that in patients with stable COPD lower oxygen content of blood as a measure of peripheral oxygen supply, lower exercise capacity in terms of 6-MWD, and higher CRP levels were associated with reduced cognitive capacity. Furthermore, a reduction in cognitive capacity was associated with reduced disease-specific quality of life. As a potential clinical implication of this work, we suggest to screen especially patients with low oxygen content and low 6-MWD for cognitive impairment.

## Background

Previous investigations showed an association between cognitive impairment and chronic obstructive pulmonary disease (COPD) with and without hypoxemia [1, 2]. One of the potential links between both disorders is chronic inflammation, a major characteristic of COPD, which also plays an important role in the development and progression of cognitive impairment [3]. There are also shared risk factors, such as smoking, higher age, reduced physical activity and recurrent infections; moreover COPD patients often suffer from comorbidities, e.g. diabetes [4], cardiovascular diseases [5], sleep apnoea [6], hyperlipidemia [7] and depression [8], which may affect cognitive function.

Regarding cognitive function there is a wide range from mild impairment to manifest dementia. A higher prevalence of cognitive impairment has been shown in COPD compared to non-COPD [3, 9], and a moderate or severe dysfunction was identified in 61% of hypoxemic COPD patients [10], whereas the prevalence of severe dysfunction in terms of dementia in the general population is around 12% [11]. COPD is characterized by chronic airflow limitation. The link between lung function and cognition has been investigated in studies of lung-healthy elderly subjects [2]. Indeed, a reduced lung function was associated with worse cognitive performance and an increased risk of hospital admission due to dementia [12].

In line with this, the majority of studies in COPD observed reduced attention, memory and learning functions [13]. More severe COPD defined by the use of long term oxygen therapy or reduced physical activity was associated with more pronounced loss of cognitive capacity [14]. Conversely, decreased cognitive capacity also appears adversely affecting COPD outcomes, due to an increased hospitalisation rate, reduced quality of life and ability of disease management [2, 15, 16]. In view of the data elucidating single aspects, the aim of the study was to provide a comprehensive analysis of the relationship between cognitive function and important COPD outcome parameters and to identify independent risk factors for cognitive impairment. This is relevant, as the multiple relationships raise the question of their relative contribution, which can be answered via path analysis, as demonstrated in previous studies [17–20]. We therefore applied this method to quantify the direct and indirect effects of COPD characteristics on cognitive function using the screening instrument DemTect for the evaluation of cognitive impairment. As an integrative outcome measure the SGRQ was included. For this purpose, we used the data of the large and well-characterized COPD cohort COSYCONET (**CO**PD and **Sy**stemic **Co**nsequences-Comorbidities **Net**work).

## Methods

### Study population

The present analysis used the baseline data (visit 1) of the German COPD cohort COSYCONET (*n* = 2741), which is a multi-center study focusing on the role of comorbidities in COPD [21]. From all patients recruited into COSYCONET, we included only those of GOLD grades 1–4 and GOLD groups A-D, according to the modified Medical Research Council dyspnoea scale (mMRC) [22], with complete data on forced expiratory volume in 1 s (FEV_1_), forced vital capacity (FVC), age, gender, body-mass index (BMI), pack years, smoking status, C-reactive protein (CRP), 6-min walk distance (6-MWD) and DemTect score. This resulted in a subset of *n* = 1969 out of *n* = 2741 patients. The COSYCONET study has been approved by the ethical committees of all study centers, and all patients gave their written informed consent [21].

### Assessments

The study protocol of COSYCONET and the methods have already been described elsewhere [21]. Assessment of comorbidities including cognitive impairment was based on the patients’ reports of a physician-based diagnosis. Some comorbidities that were used for sensitivity analyses were additionally evaluated using a combination of patients’ reports and disease-specific medication [23]; data on Il-6, Il-8 and TNF assessed by standard laboratory procedures were also used for sensitivity analyses. Lung function data comprised FEV_1_, FVC in percent predicted, and their ratio, but only FVC was included in the path analysis, as it was superior to the other parameters. Predicted values of spirometry were taken from the Global Lung Initiative (GLI) [24]. The values of PaO_2_, PaCO_2_, pH and SaO_2_ were determined from arterialized capillary blood from the earlobe, following standardised operating procedures in all study centers [21]. The oxygen content (CaO_2_) was derived via the formula: CaO_2_ = (1.34 * Hb * SaO2) + (0.0031 * PaO_2_) [25].

To quantify the impact of COPD on several dimensions of quality of life, the St. George’s Respiratory Questionnaire (SGRQ) was used [26, 27]; based on the regression analyses, only the impact score was included in the path analysis model. For the detection and quantification of early cognitive impairment the screening test DemTect [28] was used. The DemTect is carried out in the form of an oral and written interview and the patient’s performance is recorded by the examiner on a test sheet. In COSYCONET, the test was carried out together with the study nurse during the regular study visits. It contains five tasks concerning the functions of verbal memory, word fluency, intellectual flexibility and attention. The test does not take a long time (8–10 min).

Content:

- Listen and repeat word list
- Convert numbers and numerals
- Supermarket task (listing 30 goods available in a supermarket)
- Repeat number sequences backwards
- Repeat word list

The raw values of the test are recoded into test values (separated at the age of 60 years) and then summed up so that the final test values are expressed regardless of age. The scale ranges from 0 to 18 points: Values of 13 points and upwards indicate adequate cognitive performance, between 9 and 12 points mild cognitive impairment, and dementia for values equal to or below 8. According to the design, the test values should not only be independent of the age-appropriate decline in cognitive abilities, but also independent of the level of education [28].

### Statistical analysis

Data are presented as numbers and percentages, or mean values and standard deviations (SD). Comparisons between GOLD groups A-D were performed by univariate analysis of variance (ANOVA), or by chi-square-tests in case of categorical variables; for CRP the Kruskall-Wallis-H-test was used due to a heavily skewed distribution. The associations between variables were evaluated by multiple linear and logistic regression analyses comprising one dependent and multiple independent variables. CRP was included after logarithmic transformation to account for the skewness of the distribution. In these analyses, age, gender and smoking status were always included as confounders; BMI and pack years did not appear to be statistically relevant.

In the subsequent path analysis, appropriately adjusted values were used to take into account the dependence on the common risk factors and avoid trivial associations. Path analysis is a procedure designed for the description of complex networks, particularly the quantification of direct effects of one onto another variable, as well as indirect effects mediated via another variable [29]. This differentiation has been found to be informative for the description of complex associations in COPD [17–20]. In general, path analysis models require input from previous regression analyses and pathophysiological considerations to identify a valid and plausible structure, excluding models that do not describe the data adequately [29]. All analyses were performed using SPSS Statistics 23 (IBM Corp., Armonk, NY, USA) and AMOS (IBM Corp., Armonk, NY, USA). The criterion of generalized least squares estimation (GLS) was used, whereby the goodness of fit was described by chi-square statistics, comparative fit index (CFI) and root mean square error of approximation (RMSEA). CFI values ≥0.95 and RMSEA values ≤0.05 indicate a good fit. The chi-square statistics indicate the deviation from the path analysis model and is acceptable if *p* ≥ 0.05, however, its usefulness is limited in very large data sets. The level of statistical significance for all analyses was assumed for *p* < 0.05.

## Results

### Study population

Overall, 1969 patients of GOLD grades 1 to 4 had complete data of all variables used in the regression and path analyses, as well as GOLD ABCD grouping and smoking history. Table 1 shows the patients’ characteristics stratified for the GOLD groups A-D and age. For patients under 60 years of age there were significant differences between groups A-D for all parameters except for gender, pack years, DemTect score and the diagnosis of cognitive impairment. For patients of at least 60 years of age, again most of the parameters showed significant differences between GOLD groups except for gender, DemTect score and the diagnosis of cognitive impairment. Table 2 shows the gender distribution of the different categories of cognitive functioning according to DemTect scores.

**Table 1:** Baseline characteristics of the subgroups under and above 60 years stratified according to GOLD groups

	Patients < 60						Patients > = 60						Total	
GOLD (mMRC)	GOLD A	GOLD B	GOLD C	GOLD D	Total	*p*	GOLD A	GOLD B	GOLD C	GOLD D	Total	*p*	*n* = 1969	*p*
	*n* = 207	*n* = 91	*n* = 86	*n* = 128	*n* = 512		*n* = 582	*n* = 389	*n* = 180	*n* = 306	*n* = 1457			
Gender (m/f)	123/84	46/45	48/38	68/60	285/227	0.478	375/207	257/132	118/62	181/125	931/526	0.254	1216/753	0.205
Age (y)	53.7 ± 4.7	54.0 ± 4.4	53.1 ± 5.1	54.8 ± 3.7	53.9 ± 4.5	0.049	68.7 ± 5.6	69.2 ± 5.9	68.4 ± 5.4	68.3 ± 5.4	68.7 ± 5.6	0.192	64.9 ± 8.4	< 0.001
BMI (kg/m^2^)	26.3 ± 4.9	28.3 ± 6.5	26.0 ± 4.7	26.6 ± 6.4	26.7 ± 5.6	0.022	26.3 ± 4.5	27.4 ± 5.3	25.9 ± 4.3	27.1 ± 5.8	26.7 ± 5.0	< 0.001	26.7 ± 5.2	< 0.001
Pack years	38.9 ± 26.8	47.3 ± 33.0	41.9 ± 28.3	45.3 ± 36.3	42.5 ± 30.9	0.124	51.2 ± 36.7	54.4 ± 37.7	47.1 ± 35.5	52.3 ± 39.4	51.8 ± 37.4	0.203	49.3 ± 36.0	0.027
DemTect Score	15.6 ± 3.0	16.1 ± 3.1	16.2 ± 2.8	15.6 ± 3.2	15.8 ± 3.0	0.314	16.5 ± 2.7	16.3 ± 2.8	15.9 ± 2.9	16.1 ± 3.0	16.3 ± 2.8	0.039	16.2 ± 2.9	0.169
FEV_1_% predicted	59.2 ± 17.0	44.1 ± 15.4	51.8 ± 17.6	41.1 ± 16.4	50.7 ± 18.3	< 0.001	62.2 ± 18.1	49.6 ± 16.5	55.4 ± 16.0	44.3 ± 14.8	54.3 ± 18.2	< 0.001	53.4 ± 18.3	< 0.001
FVC % predicted	84.8 ± 17.6	71.2 ± 16.1	80.7 ± 15.9	67.2 ± 18.3	77.3 ± 18.8	< 0.001	86.5 ± 18.1	75.9 ± 17.1	82.8 ± 16.5	69.6 ± 17.6	79.7 ± 18.8	< 0.001	79.1 ± 18.8	< 0.001
CaO_2_ ml/100 ml	19.2 ± 1.6	18.9 ± 1.6	19.1 ± 1.5	18.7 ± 1.7	19.0 ± 1.6	0.026	18.9 ± 1.7	18.5 ± 1.8	18.4 ± 1.6	18.1 ± 1.8	18.6 ± 1.8	< 0.001	18.7 ± 1.7	< 0.001
SGRQ sum score	31.7 ± 16.0	52.7 ± 15.4	38.4 ± 15.5	61.1 ± 13.4	43.9 ± 19.6	< 0.001	29.1 ± 14.9	51.1 ± 15.5	38.9 ± 16.9	59.2 ± 15.5	42.5 ± 19.8	< 0.001	42.9 ± 19.7	< 0.001
PHQ-9 sum score	5.7 ± 4.2	9.0 ± 5.4	7.3 ± 4.2	10.1 ± 5.0	7.7 ± 5.0	< 0.001	4.1 ± 3.4	6.6 ± 4.5	5.3 ± 3.9	8.0 ± 5.1	5.7 ± 4.5	< 0.001	6.2 ± 4.7	< 0.001
6-MWD (m)	488 ± 85.4	404.6 ± 83.6	466.4 ± 98.8	385.7 ± 98.8	444.1 ± 101.1	< 0.001	460.9 ± 79.1	372.1 ± 97.8	447.6 ± 91.5	339.4 ± 106.2	410.1 ± 105.3	< 0.001	418.9 ± 105.2	< 0.001
CRP (Median/ Interquartile range)	0.31/0.46	0.40/0.73	0.46/0.42	0.50/0.68	0.41/0.43	0.006	0.36/0.42	0.50/ 0.59	0.50/0.47	0.50/0.59	0.45/0.50	0.003	0.43/0.49	< 0.001
Diagnosis of cognitive impairment (%)	8 (3.9)	9 (9.9)	4 (4.7)	9 (7.0)	30 (5.9)	0.194	26 (4.5)	21 (5.4)	5 (2.8)	22 (7.2)	74 (5.1)	0.149	1865/104	0.063

The table shows mean values and standard deviations or absolute numbers, in case of the diagnosis of cognitive impairment additionally percentages. Column 7 and 13 show the *p*-values of comparisons between patients of the respective GOLD groups A-D (univariate ANOVA or chi-square-tests in the case of categorical variables). Due to the skewness of distribution of CRP values, median values and interquartile range have been chosen for presentation. The comparison between groups was performed with log-transformed values

**Table 2:** Gender distribution of the different categories of cognitive functioning according to DemTect scores

Gender	Dementia	Mild cognitive impairment	Healthy	Total
	*n* = 31	*n* = 205	*n* = 1733	*n* = 1969
Male (%)	25 (80.6)	169 (82.4)	1022 (59.0)	1216
Female (%)	6 (19.4)	36 (17.6)	711 (41.0)	753

The table shows absolute numbers as well as percentages of men and women in different DemTect categories. The DemTect scale ranges from 0 to 18 points: Values of 13 points and upwards indicate adequate cognitive performance (healthy), between 9 and 12 points mild cognitive impairment, and dementia for values equal to or below 8

### Dependence of the DemTect score on functional and clinical parameters

To determine the statistical dependence of the DemTect score on influencing variables, we performed multiple linear and logistic regression analyses, including the functional and clinical variables listed in Tables 3 and 4 and the common risk factors. Oxygen content was superior to the use of PaO_2_, which was not significantly related to the DemTect score. FVC, FEV_1_, residual volume (RV), total lung capacity (TLC), the ratio RV/TLC and intrathoracic gas volume (ITGV) were not significantly related to the DemTect; in Tables 3 and 4, FVC is shown, as it showed the strongest correlation with the DemTect among all lung function parameters. This was also true in the subsequent establishment of the path analysis model in which FVC was superior to all other lung function parameters.

**Table 3:** Dependence of DemTect score in original form on clinical and functional parameters

	Unstandardized regression coefficient	SEM	95% Confidence Interval		*p*-value
			Lower limit	Upper limit	
Age ≥ 60 y *	1.765	0.222	1.330	2.199	< 0.001
Female	1.243	0.138	0.971	1.515	< 0.001
Age [y]	−0.079	0.012	−0.103	− 0.056	< 0.001
Current smoker	0–.444	0.138	−0.733	− 0.156	0.003
Diagnosis of CI	−0.617	0.279	−1.164	−0.069	0.027
mMRC	0.197	0.091	0.019	0.376	0.030
SGRQ impact score	−0.009	0.004	−0.017	− 0.002	0.015
6-MWD [m]	0.002	0.001	0.000	0.004	0.010
log_10_CRP [mg/100 mL]	− 0.350	0.021	−0.583	− 0.116	0.003
FVC [%predicted]	0.006	0.004	−0.002	0.013	0.135
CaO_2_ [mL/100 mL]	0.090	0.039	0.014	0.167	0.020

The table shows the results of multivariable linear regression analyses in terms of the unnormalized regression coefficients as well as 95%-confidence intervals. The level of significance was set at *p* < 0.05. *Indicator variable with values 0 and 1 for ages < and ≥ 60 years of age, respectively. CRP values were included as log_10_-transformed values, therefore the respective coefficient must be interpreted as change in the respective DemTect score for a tenfold increase in CRP level (log_10_(10) = 1). CI = cognitive impairment. When repeating the same analysis while including the activity and symptom scores of the SGRQ, these turned out to be not statistically significant predictors (*p* = 0.247 and *p* = 0.127, respectively), while the impact score remained significant (*p* = 0.0013)

**Table 4:** Dependence of the modified DemTect score on clinical and functional parameters

	Unstandardized regression coefficient	SEM	95% Confidence Interval		*p*-value
			Lower limit	Upper limit	
Age ≥ 60 y *	0.003	0.225	−0.438	0.445	0.989
Female	1.352	0.141	1.076	1.628	< 0.001
Age [y]	−0.088	0.012	−0.112	− 0.064	< 0.001
Current smoker	−0.373	0.149	−0.666	− 0.081	0.012
Diagnosis of CI	−0.731	0.284	−1.287	−0.175	0.010
mMRC	0.183	0.092	0.002	0.364	0.047
SGRQ impact score	−0.009	0.004	−0.017	− 0.002	0.017
6-MWD [m]	0.002	0.001	0.001	0.004	0.007
log_10_CRP [mg/100 mL]	−0.317	0.121	−0.554	− 0.079	0.009
FVC [% predicted GLI]	0.005	0.004	−0.003	0.013	0.187
CaO_2_ [mL/100 mL]	0.110	0.039	0.033	0.188	0.005

The table shows the results of multivariable linear regression analyses in terms of the unnormalized regression coefficients as well as 95%-confidence intervals. The level of significance was set at *p* < 0.05. *Indicator variable with values 0 and 1 for ages < and ≥ 60 years of age, respectively. CRP values were included as log_10_-transformed values, therefore the respective coefficient must be interpreted as change in the respective DemTect score for a tenfold increase in CRP level (log_10_(10) = 1). CI = cognitive impairment. When repeating the same analysis while including the activity and symptom scores of the SGRQ, the activity score turned out to be not statistically significant (*p* = 0.120), while the symptom score was only borderline significant (*p* = 0.048) and the impact score remained significant (*p* = 0.000313)

The DemTect uses an age-dependent scoring algorithm to transform raw scores for age groups < 60 and ≥ 60 years. We therefore included a categorical variable indicating the two different age groups. The result of the regression analysis using the original DemTect score is shown in the Table 3. The categorical variable was highly statistically significant, indicating an upward step at the age of 60 years. Since this sudden change seemed implausible and the inclusion of the categorical variable might disturb analyses, we defined a revised DemTect score by calculating both age-dependent transformed scores independent of age and averaging the two scores. The results of the regression analysis using this modified DemTect score are shown in Table 4. All variables identified as predictors using the original DemTect score were also significant predictors of the modified score, while the categorical value describing the two age groups was not significant. To avoid the complication from the categorical variable, we used the modified DemTect score in all subsequent analyses.

**Fig. 1:**
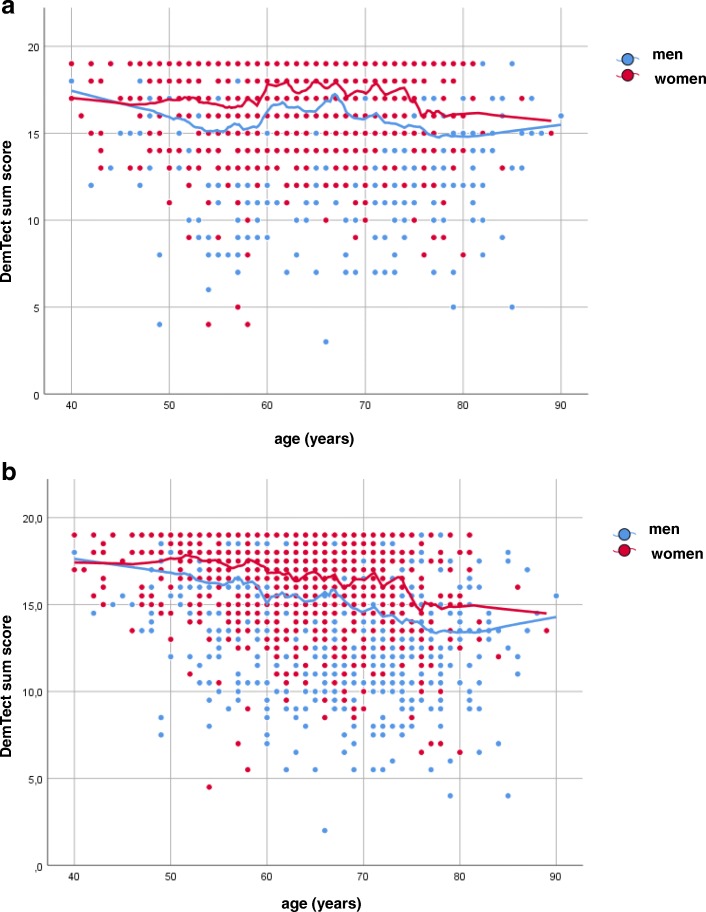
**a**. Scores of the DemTect Test versus age for men (blue circles) and women (red circles) separately as evaluated according to the original algorithm. **b**. Scores of the DemTect Test versus age for men (blue circles) and women (red circles) separately as obtained by averaging the two different evaluation algorithms that were applied independent of age

For illustration, Fig. 1a shows the DemTect score evaluated by the original algorithm versus age, with an upwards step at the age of 60 years. Fig. 1b shows the results obtained by averaging the two evaluation algorithms independent of age, indicating the absence of such a step. When using the original DemTect score, 25 men and 6 women showed values < 9 points, and 194 and 42 < 13 points, respectively. When using the modified score, 42 men and 11 women showed values < 9 points, and 286 and 70 < 13 points, respectively. As expected, the modified score affected the categorization of abnormalities, but this was irrelevant for the association analyses.

**Fig. 2:**
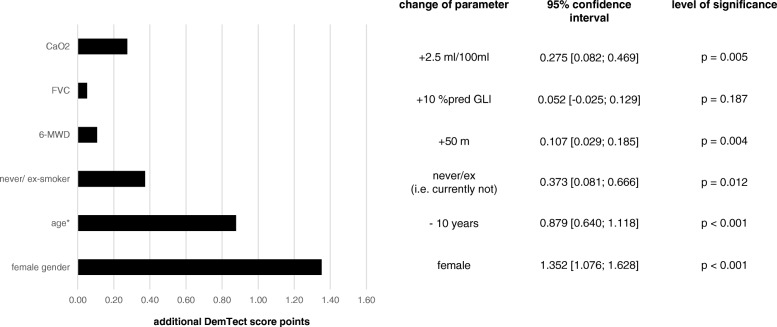
Estimated changes in the DemTect score for defined changes in predictors according to the regression coefficients given in table 2b

To illustrate the effect of influencing parameters on the modified DemTect score, the respective changes as computed according to Table 3 are shown in Fig. 2.

### Dependence of the SGRQ score on functional and clinical parameters

Regarding the dependence of the total SGRQ score and its components (activity, impact, symptoms) on influencing variables, we again performed multiple regression analyses including the variables listed in Table 5. Gender, the diagnosis of cognitive impairment, age, 6-MWD, FVC %predicted, mMRC score and the modified DemTect score were significant predictors of the impact SGRQ score (*p* < 0.05, Table 5), whereas for the activity and symptoms components and total score there were less associations. To avoid problems from collinearity and to keep the model parsimonious, we selected the impact score for the path analysis, excluding the other two domains and the total domain that was strictly linearly dependent on the three domains [26].

**Table 5:** Dependence of the impact score of the SGRQ on clinical and functional parameters

	Unstandardized regression coefficient	SEM	95% Confidence Interval		*p*-value
			Lower limit	Upper limit	
Female	−1.884	0.822	−3.496	−0.271	0.022
Age [y]	−0.249	0.048	−0.342	−0.156	< 0.001
Current smoker	0.122	0.856	−1.556	1.801	0.886
Diagnosis of CI	70.029	1.618	3.856	10.201	< 0.001
mMRC	90.996	0.478	9.059	10.933	< 0.001
6-MWD [m]	−0.044	0.004	−0.052	−0.035	< 0.001
log_10_CRP [mg/100 mL]	0.024	0.693	−1.335	1.383	0.973
FVC [% predicted GLI]	−0.060	0.022	−0.105	− 0.016	0.007
CaO_2_ [mL/100 mL]	−0.182	0.226	−0.626	0.262	0.422
Modified DemTect score	−0.309	0.129	−0.562	−0.055	0.017

The table shows the results of multivariable linear regression analyses in terms of the unnormalized regression coefficients as well as 95%-confidence intervals. The level of significance was set at *p* < 0.05. *Indicator variable with values 0 and 1 for ages < and ≥ 60 years of age, respectively. CRP values were included as log_10_-transformed values, therefore the respective coefficient must be interpreted as change in the respective DemTect score for a tenfold increase in CRP level (log_10_(10) = 1). *CI* Cognitive impairment

### Path analysis

**Fig. 3:**
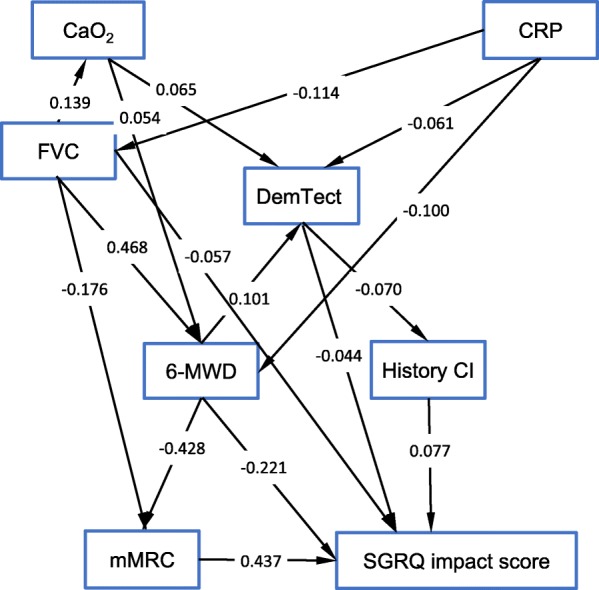
Path analysis model of the major variables evaluated in the present study

To understand the role of the DemTect score, we used path analysis. Age, gender and smoking status turned out to be correlated with all variables. In the path analysis we therefore used values adjusted for these predictors in order to find a parsimonious model not complicated by trivial links. The model was built consecutively using the results of Tables 3 and 4 as well as results of additional regression analyses of the variables on each other. The final model is shown in Fig. 3 and Table 6. It fitted the data well with a CFI of 0.988, a RMSEA of 0.020 (90%CI: 0.002; 0.033) and a Chi-square value of 21.28 at 12 degrees of freedom (*p* = 0.046).

**Table 6:** Results of the path analysis model

Regression	Estimate	S.E.	C.R.	Standardized.	*P*
FVC ← CRP	−4.059	.799	−5.078	−.114	*p* < 0.001
CaO_2_ ← FVC	.012	.002	6.180	.139	*p* < 0.001
6-MWD ← FVC	2.564	.109	23.532	.468	*p* < 0.001
6-MWD ← CaO_2_	3.471	1.263	2.749	.054	.006
6-MWDs←CRP	−19.386	3.845	−5.041	−.100	*p* < 0.001
DemTect←CaO_2_	.115	.040	2.903	.065	.004
DemTect←CRP	−.326	.121	−2.681	−.061	.007
DemTect←6-MWD	.003	.001	4.417	.101	*p* < 0.001
History of CI ← DemTect	−.005	.002	−3.075	−.070	.002
mMRC←6-MWD	−.004	.000	−19.576	−.428	*p* < 0.001
mMRC←FVC	−.008	.001	−8.072	−.176	*p* < 0.001
SGRQ (impact) ← DemTect	−.315	.129	−2.450	−.044	.014
SGRQ (impact) ← FVC	−.062	.022	−2.783	−.057	.005
SGRQ (impact) ← History of CI	7.061	1.626	4.341	.077	*p* < 0.001
SGRQ (impact) ← mMRC	9.988	.479	20.868	.437	*p* < 0.001
SGRQ (impact) ← 6-MWD	−.044	.004	−9.952	−.221	*p* < 0.001

The panel refers to the directed arrows (regression terms) depicted in Fig. 3, whereby the left part lists the arrows shown in this figure. The right part shows the results of the corresponding statistical tests. The first column of the right part shows the non-standardized estimate of the respective regression coefficient, the second column the standard error (S.E.) of this coefficient, the third column the ratio of these two values (critical ratio, C.R.) which is used for significance testing. The fourth column shows the standardized estimates of the regression coefficients shown in the first column. The last column shows the significance level based on the generalized least squares (GLS) procedure of AMOS. In the path analysis model, CRP values were logarithmically transformed (log10), in order to account for the skewness of data and obtain a distribution closer to normal. For abbreviations of symbols see text

The impact score turned out to be directly dependent on the DemTect results but also on the diagnosis of cognitive impairment. This diagnosis was associated with the DemTect results, indicating the validity of both variables. As a result, there was a direct and an indirect contribution of the DemTect to the impact score. The DemTect result itself, was dependent on oxygen content, CRP and 6-MWD, consistent with the results of the regression analyses. In the path analysis, the mMRC turned out to be only indirectly associated with the DemTect, in contrast to the regression analysis, revealing the differentiation between direct and indirect links. Still, however, it was directly linked to the SGRQ impact component, 6-MWD and FVC. FVC was related to 6-MWD and oxygen content. It is noteworthy that FVC could not be replaced by FEV_1_, FEV_1_/FVC, RV, or ITGV since this resulted in the loss of statistical significance of associations. Regarding RV/TLC, however similar results as for FVC were obtained, with slightly weaker relationships to CaO_2_ and the impact score than for FVC. When the mMRC was replaced by the SGRQ symptoms score, all single relationships remained significant but the overall model fit was inferior (chi-square 36.45, *p* < 0.001) thereby underlining the superiority of the mMRC within the network of variables analyzed.

### Sensitivity analyses

These results indicated that the DemTect score could be consistently embedded into the network of functional and clinical measures that were directly or indirectly linked to it. In order to reveal whether the relationship between the other variables was robust against the inclusion of the DemTect, the path analysis was repeated while omitting the DemTect. All associations remained significant. The chi square was 19.78, with 10 degrees of freedom (*p* = 0.031). The fact that the structure remained statistically significant after removing the DemTect was consistent with the fact that the DemTect had only a small explanatory value on the impact score, beyond the effects of the other variables (see Fig. 3). Moreover, we computed a model omitting the diagnosis of cognitive impairment as it could be argued that this diagnosis requires specific expertise from the treating doctors. Again, the model was virtually unchanged, in particular the regression coefficient from DemTect to impact score (standardized value − 0.048). Furthermore, the relationship between cognitive function and cytokine levels (Il-6, Il-8, TNF) is often discussed, we therefore assessed their relationship to the DemTect score or other variables of the model, either including or excluding CRP. In none of these models the cytokines were significantly related to other variables including the DemTect score.

## Discussion

The present analysis had the aim to develop an integrative picture of cognitive function in relation to major COPD characteristics, taking into account their mutual relationships. For the quantification of cognitive function, we used the DemTect score, a sensitive test for mild changes preceding overt dementia [28]. DemTect values were in the normal range in most patients. We confirmed a number of associations identified in previous studies and in particular found a reduction of oxygen content of peripheral blood to be associated with cognitive decline in stable COPD. Moreover, COPD-specific quality of life was independently associated with a reduction in cognitive function in addition to the impact of COPD-specific predictors. The DemTect score, when plotted against age, showed a discontinuity at the age of 60, as a consequence of the different evaluation algorithms below and above that age. Whereas this may be adequate for clinical applications, it introduced an unnecessary complication into statistical analyses, and consequently we defined a modified, average score that exhibited a smooth course versus age. The modified score showed the same or better associations with the other variables compared to the original score, while the number of patients with scores considered as clinically suspicious was higher, reflecting the fact that the modification mainly affected patients with higher age. For the purpose of association analyses the modified score, however, appeared to be superior to the original score.

Cognitive impairment is relevant in patients with COPD, as it can affect the self-management of the disease, for example by forgetting the recommended vaccinations, inadequate intake or non-intake of medication, reduction of the effectiveness of pneumological rehabilitation including breathing therapy at home, and as a result having detrimental effects of the therapeutic measures forming the basis for COPD therapy. The prevalence of DemTect results indicative of mild cognitive impairment was lower in our COPD population than in previous studies using other screening tools [11]. In addition to differences between the tools, the fact might contribute, that COSYCONET incorporates a rather comprehensive assessment program favouring the underrepresentation of patients with cognitive impairment. The smaller range could reduce the statistical power, nevertheless we found many associations which were consistent with those reported in the literature. This renders it unlikely that our results were significantly affected by the low prevalence of suspicious DemTect findings. The results were also robust against the exclusion of the reported diagnosis of cognitive impairment, which is likely to refer predominantly to patients with lower DemTect scores; indeed, the group of patients with the respective history (*n* = 104) showed significantly lower scores than the group without history. We found no significant differences in the DemTect scores across the GOLD groups A-D based on symptoms and exacerbations [22]. The fact that the DemTect scores in path analysis model were not directly linked to the mMRC scores, is in line with the results from Table 1 showing no differences in the DemTect scores between GOLD groups A-D. Previous studies showed associations between exacerbations and cognitive decline, but cognitive impairment improved after discharge from hospital following exacerbation [9, 30]. In COSYCONET, we included patients with stable COPD and did not expect differences in cognitive impairment across GOLD groups A-D. The clinical interpretation could be that particularly in stable COPD, symptoms, respiratory function and exercise capacity carry different information and that the relevant relationships depend on the specific aspect studied.

Up to now, there is no common agreement on the definition of cognitive impairment in COPD patients, and the variety of definitions renders comparisons across studies difficult [9]. The Mini-Mental State Examination (MMSE) is often applied for screening, but in patients with executive dysfunctions its sensitivity for mild impairment has been questioned, and in general the test is rather insensitive to minor cognitive changes [11, 31, 32]. Previous investigations revealed that the Montreal Cognitive Assessment (MoCa) was more sensitive to detect minor cognitive deficits than the MMSE [33–35], furthermore it was shown that the MoCa was superior to MMSE in COPD patients [11]. At the time when COSYCONET was planned, the DemTect was considered as the most sensitive test for mild impairment. It was especially designed to detect mild cognitive impairment (MCI) and dementia in early stages of Alzheimer’s dementia (AD), being powerful in the detection of Alzheimer’s dementia (sensitivity 100%) and mild cognitive impairment (sensitivity 80%) [28] . A study comparing the effectiveness of tests for the identification of MCI and mild dementia, showed that the DemTect achieved the best results, with a sensitivity of 90% for MCI and perfect group discrimination for mild dementia. We therefore think, that the DemTect was well-suited as a screening tool for mild cognitive impairment in our population of patients with stable COPD [36].

To the best of our knowledge there are no studies comparing the DemTect with MMSE and MoCa in COPD, but there are data in patients without COPD showing comparable sensitivity of DemTect and MoCa in the detection of minor cognitive change [37, 38].

Former investigations found that more severe COPD was associated with a higher prevalence of cognitive impairment [39, 40]. COPD patients with hypoxemia showed a prevalence of up to 77% [10], whereas mild hypoxemia was associated with impairments comparable to healthy subjects [41]. Moreover, neuroimaging studies identified associations between decreased perfusion and increased cognitive impairment in hypoxemic COPD patients [42]. Many of these studies had limitations, e.g. methodological problems such as diagnostic uncertainty in the classification of COPD, or uncertainties in the detection of mild cognitive impairment by the use of MMSE, or small sample size [40]. The use of the DemTect as a very sensitive tool also for mild cognitive impairment, and the broad panel of validated lung function data available in COSYCONET are therefore superior to these investigations. Nevertheless, these findings are in accordance with our result that higher CaO_2_ was associated with higher DemTect scores (see Tables 3 and 4). The path analysis revealed that this association was not explained by confounding from other variables such as lung function or risk factors, such as age and gender. Furthermore, our investigations revealed that the oxygen content was a much stronger predictor of DemTect scores than oxygen partial pressure and oxygen saturation. The oxygen supply of the organs is restricted by the number of oxygen molecules and by perfusion of the peripheral arteries. The use of oxygen content is superior to PaO_2_ or oxygen saturation as it takes into account compensation mechanisms to avoid organ hypoxemia like polycythemia, changes in 2,3-diphosphoglycerateconcentration and increase in oxygen-resistant isoenzymes of the respiratory chain [43]. The higher importance of CaO_2_ compared to PaO_2_ has specifically been shown for cerebral perfusion [44].

The magnitude of the dependence on CaO_2_ was remarkable. An increase by 2.5 ml per 100 ml of blood corresponded to an increase of the modified DemTect score by nearly 0.3 points. Among the functional parameters, this appeared to have the strongest effect although the dependence on age and gender was even stronger as illustrated in Fig. 2. This observation also underlined the importance of adjusting for age, gender and smoking status in the path analysis model.

In line with our results, the risk of dementia and physical activity have been found to be inversely correlated [45, 46]. The observed association between better 6-MWD and better memory function in a small geriatric population without COPD [47] also supports our results. Moreover, the association with CRP is consistent with previous findings regarding elevated CRP levels [47]. It should be noted that COSYCONET patients are studied in a stable condition [21] and that therefore an elevated CRP may be caused by a chronic systemic inflammation. Again, in the path analysis model the association with CRP was robust in the presence of several confounders. CRP might be related to cytokine activation and chronic inflammation, which are known to play a role in the progression of neurodegenerative diseases. In our study however, neither IL-6, nor IL-8, nor TNF showed a significant association with the DemTect score or any other variable. This might reflect the fact that we used values adjusted for major risk factors (age, gender, smoking status) but also the fact that systemic inflammation was dominated by the lung disease and potential contribution from neurodegenerative disease played a minor role.

In our large population of patients, the DemTect score showed a clear-cut discontinuity at the age of 60 years corresponding to the switch in the evaluation in patients of age ≥ 60 years. We do not question that this switch is sensible for diagnostic purposes, however its magnitude was fairly large compared to the mean range of the DemTect over age (see Fig. 1a, Table 3). It is unlikely that the discontinuity is specific for patients with COPD, and we therefore used a modified, averaged score, to avoid statistical complications and a loss of statistical power. These disadvantages became apparent when comparing the regression results (Tables 3 and 4).

Importantly, the associations found in the path analysis were consistent with previous data on cognitive function in COPD but allowed to identify direct and indirect effects, taking into account all major single determinants described previously. The DemTect was directly influenced by COPD severity, especially oxygen content and 6-MWD, and had an independent direct effect on the impact domain of the SGRQ, i.e. a lower DemTect score was associated with lower disease-specific quality of life. The three dimensions of the SGRQ are not equivalent. For example, in the recent analysis on cardiac comorbidities only the activity dimension turned out to be relevant [48]. In the present study, only the impact score showed significant and consistent associations with cognitive function, but not the total score comprising the two other dimensions. The unstandardized regression coefficient from DemTect to the impact domain of SGRQ indicating an increase in this domain by 0.315 score points for an increase in cognitive function by 1 point (please note that the scales are opposite). Regarding the SGRQ impact domain, the standardized coefficients show that the direct effect of DemTect (− 0.044, Fig. 3 and Table 6) was about ten-fold stronger than the indirect effect mediated by a history of cognitive impairment (implying the product of standardized coefficients − 0.070*0.077 = − 0.005). Conversely, the link via 6-MWD (− 0.221*0.101 = − 0.022) was about half as strong as the direct link from DemTect. This shows that the direct association between cognitive impairment and disease-specific quality of life was relatively strong. The impact score covers a range of disturbances of psycho-social function, is related to respiratory symptoms, exercise capacity, breathlessness in daily life and disturbances of mood and is therefore suitable to describe broad panel of disturbances in the life of respiratory patients [26, 27].

The lung function parameter showing the highest explanatory power was not FEV_1_ but FVC, but its effect on the DemTect result was only indirect and mediated via 6-MWD. FVC as a measure of available lung volume is a marker of a restrictive lung function pattern, and more closely related to a more advanced age, which is also associated with cognitive supply. Possibly, FVC better reflects a more severe chronic lung function impairment than FEV_1_ because FVC will decrease with more marked obstruction. This was also underlined by its strong link with the oxygen content as parameter of organ oxygen supply. A different interpretation could be that the ability to perform spirometry is reduced in patients with cognitive impairment. RV/TLC, which in some respect is a complement of FVC, showed similar relations as FVC, although the relationships were not as strong. The findings obtained by omission of the DemTect score from the model in the sensitivity analysis underlined that it fitted very well into the robust relationships between the other measures. The presence of a physician-based diagnosis of cognitive impairment had an independent impact on the SGRQ impact score probably additionally reflecting the severity of the impairment, as the DemTect was still relevant upon its inclusion.

### Limitations

One of the limitations of the study is its cross-sectional, non-interventional design. Moreover, we did not have a control cohort in terms of patients without COPD. In addition, the number of patients with clinically suspicious DemTect results was low and the mean age was 65 years, i.e. lower than typical ages in patients with severe cognitive impairment. Irrespective of this, we found statistically robust associations, all of which were consistent with those described in the literature for patients with more severe cognitive impairment. As we included a variety of influencing factors, we were able to compare the relative contributions of several determinants on both the DemTect results and the social impact component of the SGRQ as major outcome variable. Comorbidities other than the diagnosis of cognitive impairment were not directly linked to the DemTect results but only to factors related to the DemTect, such as lung function oxygen content, physical activity and COPD symptoms. We therefore omitted their inclusion into the model. The presence of comorbidities was on the one hand based on the patients’ report, representing conditions known, memorized and openly reported by the patients [23]. On the other hand, we also applied a strategy to confirm self-reported diagnoses via medication as previously described by us, in order to increase the reliability of the data [23].

## Conclusions

In patients with stable COPD, cognitive capacity in terms of the DemTect score was related to major characteristics of COPD severity. Especially lower oxygen content of blood as a measure of peripheral oxygen supply, lower exercise capacity in terms of 6-MWD, and higher CRP levels were associated with reduced scores. On the other hand, respiratory symptoms and lung function, specifically FVC and RV/TLC, both of which were related to oxygen content, and cognitive capacity were determinants of quality of life, specifically the SGRQ impact score. Path analysis revealed cognitive capacity affecting quality of life, but quality of life not affecting cognitive capacity. As a potential clinical implication of this work, we suggest to screen especially patients with low oxygen content and low 6-MWD for cognitive impairment. These patients could be supported by nursing services for medication administration and a closer contact with the treating physician. As a technical observation, our results also suggest that for quantitative analyses the evaluation of the DemTect should be modified.

## Data Availability

The basic data are part of the German COPD cohort COSYCONET (www.asconet.net/) and available upon request. There is a detailed procedure for this on the website of this network. Specifically, the data can be obtained by submission of a proposal which is evaluated by the steering committee. All results to which the manuscript refers to are documented by the appropriate in the text, figures or tables.

## Abbreviations

6-MWD: 6-min walking distance
ANOVA: Analysis of variance
BMI: Body mass index
CRP: C-reactive Protein
FEV_1_: Forced expiratory volume in 1 s
FVC: Forced vital capacity
mMRC: modified Medical Research Council dyspnoea scale
QoL: Quality of life
SGRQ: St. George‘s respiratory questionnaire
